# Crystallization of Ti_33_Cu_67 _metallic glass under high-current density electrical pulses

**DOI:** 10.1186/1556-276X-6-512

**Published:** 2011-08-26

**Authors:** Dina V Dudina, Vyacheslav I Mali, Alexander G Anisimov, Oleg I Lomovsky, Michail A Korchagin, Natalia V Bulina, Maria A Neklyudova, Konstantinos Georgarakis, Alain R Yavari

**Affiliations:** 1 Institute of Solid State Chemistry and Mechanochemistry, Siberian Branch of Russian Academy of Sciences, Kutateladze str. 18, Novosibirsk 630128, Russia; 2 Lavrentiev Institute of Hydrodynamics, Siberian Branch of Russian Academy of Sciences, Lavrentiev Ave. 15, Novosibirsk, 630090, Russia; 3 Institute of Semiconductor Physics, Siberian Branch of Russian Academy of Sciences, Lavrentiev Ave. 13, Novosibirsk, 630090, Russia; 4 Science et Ingénierie des Matériaux et Procédés (SIMAP-CNRS), Institut Polytechnique de Grenoble (INPG), 1130, rue de la Piscine - 38402 Saint-Martin-d'Hères Campus, France

## Abstract

We have studied the phase and structure evolution of the Ti_33_Cu_67 _amorphous alloy subjected to electrical pulses of high current density. By varying the pulse parameters, different stages of crystallization could be observed in the samples. Partial polymorphic nanocrystallization resulting in the formation of 5- to 8-nm crystallites of the TiCu_2 _intermetallic in the residual amorphous matrix occurred when the maximum current density reached 9.7·10^8 ^A m^-2 ^and the pulse duration was 140 μs, though the calculated temperature increase due to Joule heating was not enough to reach the crystallization temperature of the alloy. Samples subjected to higher current densities and higher values of the evolved Joule heat per unit mass fully crystallized and contained the Ti_2_Cu_3 _and TiCu_3 _phases. A common feature of the crystallized ribbons was their non-uniform microstructure with regions that experienced local melting and rapid solidification.

**PACS: **81; 81.05.Bx; 81.05.Kf.

## Background

Metallic glasses are metastable materials, which crystallize when heated up to temperatures exceeding their crystallization temperature *T*_c _[1,2]. A metallic glass partially crystallized in a controlled manner contains nanocrystals in an amorphous matrix. The controlled crystallization of metallic glasses is a promising technique of developing amorphous matrix composites with improved ductility compared to monolithic metallic glasses [3,4]. However, when metallic glass fully crystallizes transforming into a mixture of intermetallics, which are very brittle, attractive mechanical properties of metallic glass and its excellent corrosion resistance are lost. The crystallization behavior of metallic glasses strongly depends on the heating rate and annealing time.

Rapid heating and short annealing durations (of the order of several seconds) can be achieved using a dc electrical current [5-7] or infrared flash annealing of metallic glasses [8]. High heating rates and extremely fast annealing/crystallization are involved in the treatments by very short electrical pulses (10^-4 ^to 10^-3 ^s) of high current density (10^7 ^to 10^9 ^A m^-2^) [9-12] and by lasers [13-15]. Rapid heating allows the metallic glass to crystallize at higher temperatures; certain crystallization stages can be bypassed. In recent years, thanks to the development of new laser experimental techniques, rapid heating of metallic glass became a part of the processes of laser-assisted amorphous coating deposition and additive manufacturing [15]. Up to date, the crystallization under pulsed heating has not been widely presented in the literature; however, recent studies show that unusual phenomena can occur when amorphous alloys are rapidly heated by a laser. LaGrange et al. [13] have shown that the kinetics of crystallization of amorphous films under extremely fast heating is very different from the kinetics observed under slow heating with nucleation and growth rates in the former case several orders of magnitude higher. In terms of microstructure, observations of the distinct features - spherulites in an amorphous matrix - locally forming under pulsed laser heating of pre-deposited powder layers are reported [15].

Electrical currents induce a variety of phenomena in amorphous materials [16-18] depending on the application mode. Due to Joule heating by the passing electrical current, metallic glass loses its thermal stability and crystallizes. As was suggested by Mizubayashi et al. [10], electrical current can induce a resonant collective motion of atoms, thereby enhancing atomic migration and facilitating crystallization at temperatures far below the crystallization temperature of the alloy.

In this work, we focus on the phase and microstructure development in the Ti_33_Cu_67 _alloy ribbons brought to successive stages of crystallization by high-density electrical pulsing. We apply a pulsed electrical current of high current density (of the order of 10^8 ^to 10^9 ^A m^-2^) to thin ribbons of the amorphous alloy Ti_33_Cu_67 _and analyze the effects of the electrical current parameters on the phase composition and microstructure of the crystallized alloy. The estimated heating rates during the electrical pulses in this work are of the order of 10^6 ^to 10^7 ^K s^-1^. So, electrical pulsing can create heating rates comparable to quenching rates used during the preparation of amorphous alloys. Electrical pulsing of high current densities and short pulse durations creates conditions of rapid heating and cooling. A sample experiencing high-density currents can fracture, fully degrade, or explode. Using extremely short pulses, certain crystallization stages can be quenched in the samples and metastable products/microstructures can be obtained. Hence, electrical pulsing makes it possible to study physical and chemical phenomena under strongly non-equilibrium conditions during heating and cooling. Even under high current densities, it is still possible to keep the sample's shape inducing partial crystallization (nanocrystallization) of the amorphous alloy.

## Results and discussion

Crystallization of the Ti_33_Cu_67 _ribbons was triggered by electrical pulses of different durations and maximum current. The mode of current decay was exponential or linear. The characteristics of the current pulses are presented in Table 1 along with the maximum current densities (calculated using the maximum current during the pulse). The Joule heat per unit mass evolved in the sample during the electric pulse can be calculated neglecting the resistivity changes. The sample mass can be expressed as

M=dl sh,

**Table 1:** Thickness of the Ti_33_Cu_67 _ribbon samples, electrical pulsing parameters, calculated current density and Joule heat evolved during the pulses

Sample	**Thickness of the ribbon**, μm	Pulse duration, μs	**Maximum current**, A	Maximum **current density^a^**, A · m^-2^	Q/m, J kg^-1^	Possible processes in the sample caused by Joule heating
N1	100	140	145	9.7 · 10^8^	5.3 · 10^4^	Heating up to approximately 480 K
N2	30	230	110	2.2 · 10^9^	3.4 · 10^5^	Heating up to the solidus temperature, partial melting
N3	40	100	284	4.7 · 10^9^	4.4 · 10^5^	Heating up to the solidus temperature, partial melting
N4	25	140	190	5.1 · 10^9^	1.0 · 10^6^	Heating up the liquidus temperature, complete melting

^a^Calculated using the maximum current

where *d *is the density of the alloy; *l *is the length; *s *is the width and *h *is the thickness of the ribbon.

The Joule heat evolved during the pulse can be expressed as follows

$$Q=\int I^{2}R\;dt.$$

The resistance of the ribbon piece can be estimated as

R=ρ l∕sh,

where *ρ *is the resistivity of the amorphous material measured to be 2.4 · 10^-6 ^Ω m.

The Joule heat evolved in the sample per unit mass is

$$Q∕M=\rho \;\int I^{2}dt∕s^{2}h^{2}d.$$

The samples N1 to N4 in Table 1 are placed in the order of increasing the maximum current density and the value of Joule heat per unit mass.

Ribbon N1 was thicker than the other samples and was set to experience the lowest current density. After the pulse, it retained its shape and did not show any visible cracks or macrodefects. On the other hand, samples N2, N3, and N4 became very brittle after electrical pulsing and contained a lot of cracks. The most severe effect was observed in sample N4. When even higher current densities were applied (not shown), the samples exploded, did not keep their wholeness and transformed into small pieces and powder particles.

**Figure 1:**
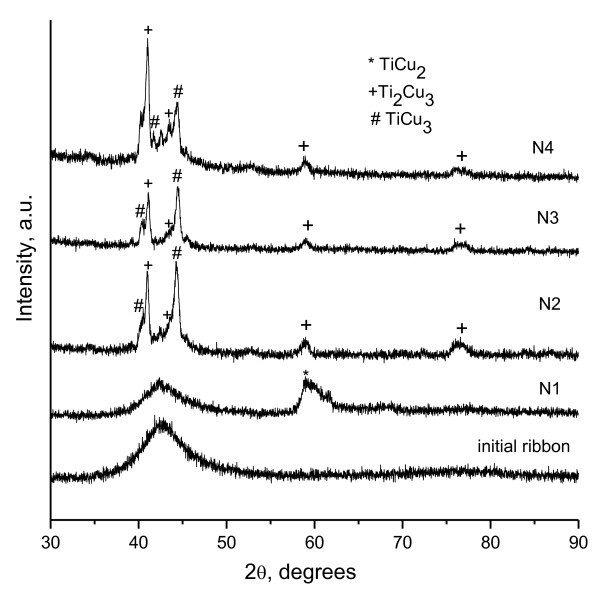
**XRD patterns of the initial Ti_33_Cu_67 _ribbon and ribbons crystallized under different electrical pulse conditions**. (see Table 1).

**Figure 2:**
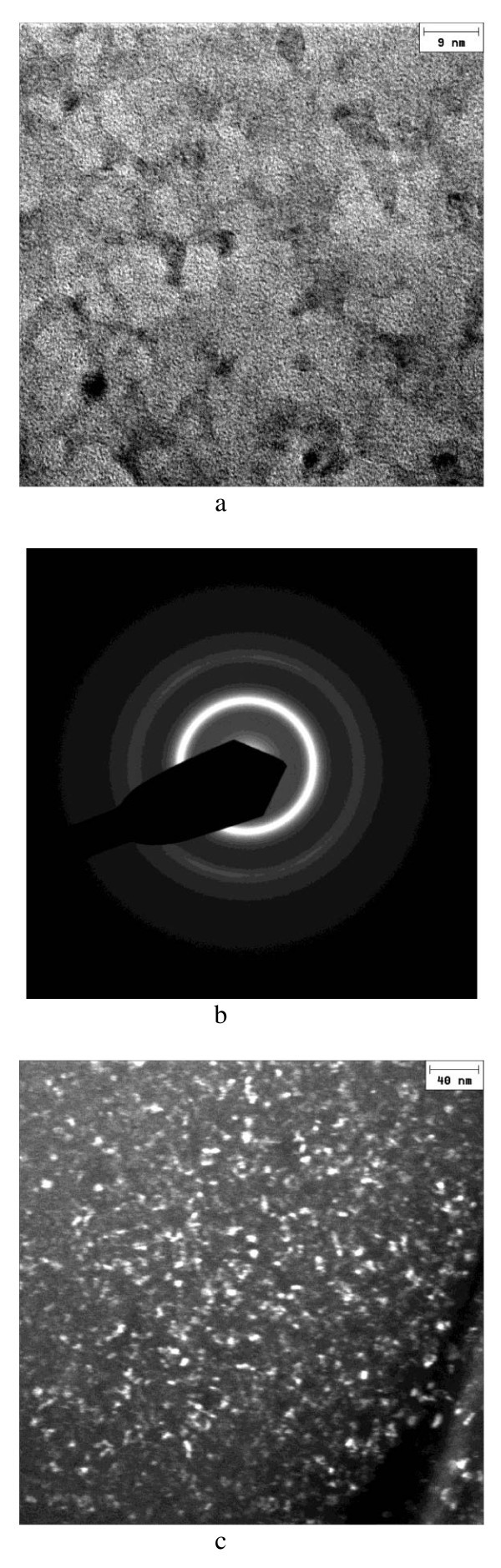
**TEM characterization of the Ti_33_Cu_67 _ribbon crystallized under a pulse of electrical current, sample N1**. **(a) **HRTEM micrograph; **(b) **selected area diffraction pattern; **(c) **dark-field electron image showing a larger area of the sample.

XRD patterns of the crystallized ribbons are shown in Figure 1 along with the pattern of the initial ribbon. The as-quenched ribbon is amorphous as is confirmed by the presence of a broad halo in the pattern. In the pattern corresponding to sample N1, we observe a halo of reduced intensity and a very broad reflection from a crystalline phase TiCu_2_, which indicates partial crystallization. Based on the shape of the XRD profile, one can anticipate the presence of very fine grains of the crystalline phase TiCu_2 _(Amm2; *a *= 4.36 Å, *b *= 7.98 Å, *c *= 4.48 Å). Figure 2a shows an HRTEM image of the crystallized alloy that has experienced partial polymorphic crystallization and shows a two-phase structure with crystalline particles of 5 to 8 nm distributed in the residual amorphous matrix. A selected area diffraction pattern is shown in Figure 2b. A larger area of the crystallized sample is shown in a dark-field image (Figure 2c). Similar microstructures can be obtained directly during casting of alloys of certain compositions [3] or can be produced through precisely controlled heating of initially amorphous alloys in *in situ *experiments using synchrotron radiation to detect the early formation of nanocrystals in an amorphous matrix [4].

The calculated Joule heat for ribbon N1 was enough only to increase the temperature of the material by 180 K from room temperature assuming the heat capacity of the Ti_33_Cu_67 _alloy *C *= 0.5·10^3 ^J kg^-1 ^K^-1^. According to Buschow [19] and Zaprianova et al. [20], the crystallization temperature of the Ti_33_Cu_67 _alloy is 700 K, which implies that during pulsing the crystallization temperature was not reached. Hence, non-thermal effects play an essential role in the crystallization processes. The crystallization of the alloy was induced by the electrical current, which is in agreement with observations of other authors [11,12]. Thus, Mizabayashi et al. [12] applied electrical pulses with a current density ranging between 1.7 · 10^9 ^and 2.7 · 10^9 ^A m^-2 ^to Zr_50_Cu_50 _amorphous ribbons triggering nanocrystallization in the sample that was not heated up to its crystallization temperature measured in the absence of current. In that case, plates of high thermal conductivity were put in contact with the ribbons during the experiment, based on which the authors considered the crystallization to be athermal and caused by electrical field only. In the present study, no special precautions were taken to dissipate heat from the sample while the current densities were of the order of 10^9 ^A m^-2^. However, it was possible to induce moderate heating and achieve partial crystallization due to the use of extremely short pulses. Also, as it will be seen below, there were a few areas in the sample, in which the temperature rose much higher than the calculated average value.

**Figure 3:**
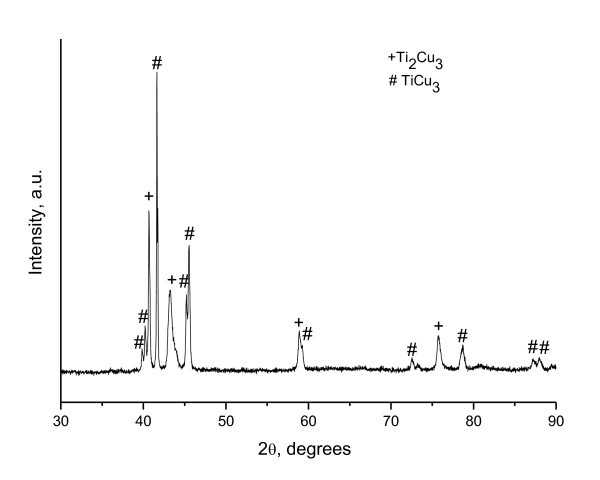
**XRD pattern of the Ti_33_Cu_67 _ribbon annealed at 773 K for 15 min**.

When higher current densities are applied to the samples, further crystallization stages are detected, as is seen from the XRD patterns of samples N2 to N4 (Figure 1). The calculated values of Joule heat are also higher compared to that of sample N1. The amorphous halo is no longer detectable in the patterns of N3, while the TiCu_2 _intermetallic phase decomposes to form intermetallic compounds indexed as Ti_2_Cu_3 _(P4/nmm; *a *= 3.13 Å, *c *= 13.95 Å) and TiCu_3 _(Pmnm; *a *= 5.45 Å, *b *= 4.42 Å, *c *= 4.30 Å). A small amount of the amorphous phase may still be present in N2 and N4. The Joule heat evolved in ribbon N2 was enough to heat the alloy up to its solidus temperature, which is 1,123 K according to [21], and partially melt it; similar processes could be expected for ribbon N3. The calculations for ribbon N4 lead to a conclusion that the sample could fully melt and then re-crystallize. Indeed, N4 was very brittle and did not retain its shape after electrical pulsing. The crystal structure of the ribbons crystallized under electrical pulses differs from that of the ribbons crystallized by conventional annealing. Figure 3 shows an XRD pattern of the ribbon annealed in vacuum at 773 K for 15 min. The ribbon is fully crystallized and contains Ti_2_Cu_3 _(P4/nmm; *a *= 3.13 Å, *c *= 13.95 Å) and TiCu_3 _(Pmmn; *a *= 5.16 Å, *b *= 4.35 Å, *c *= 4.53 Å) phases showing narrow reflections in the XRD pattern; the ratios of the XRD line intensities of the phases do not correspond to those of the ribbons crystallized by electrical pulsing. This leads to a conclusion that the crystal structure of the phases in the ribbons crystallized by pulsing is still metastable. Worth noting is the fact that the TiCu_3 _phase formed during conventional annealing has lattice parameters different from those of the TiCu_3 _phase formed during electrical pulsing (the phases were described using different JCPDS cards).

**Figure 4:**
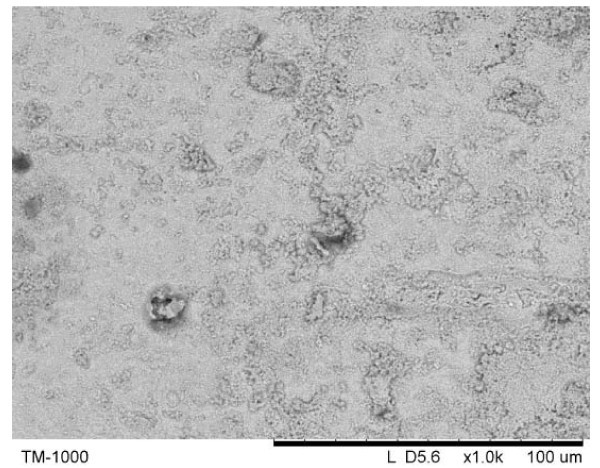
**SEM micrograph of the initial amorphous alloy ribbon Ti_33_Cu_67 _electrochemically etched in HNO_3_-CH_3_OH**.

**Figure 5:**
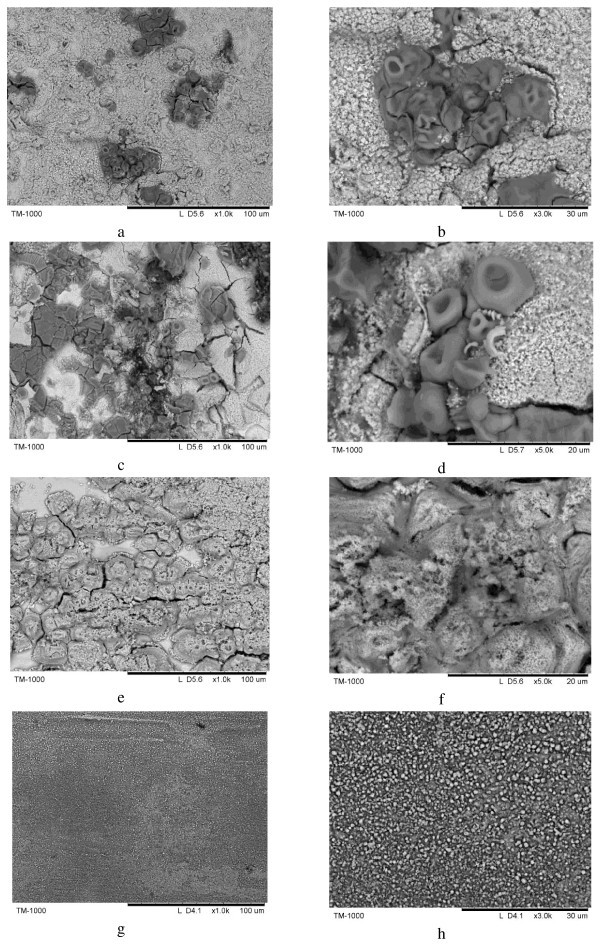
**SEM micrograph of the Ti_33_Cu_67 _ribbons electrochemically etched in HNO_3_-CH_3_OH**. **(a, b) **N1; **(c, d) **N2; **(e, f) **N3; **(g, h) **conventionally annealed at 773 K.

In order to reveal the microstructural features, the ribbons were electrochemically etched in a HNO_3_-CH_3_OH solution. Figure 4 shows a SEM image of the surface of the initial ribbon to be used as a reference when analyzing the structure of the crystallized ribbons shown in Figure 5 (two different magnifications in Figure 5 are shown for each sample in order to demonstrate unusual microstructural features and non-uniformity at different scales). A common feature of the crystallized ribbons was their non-uniform microstructure with regions that experienced local melting and rapid solidification. In N1, droplet-like featureless areas clearly indicate that melting and rapid solidification took place locally (Figure 5a,b). The surface of samples N2 (Figure 5c,d) and N3 (Figure 5e,f) subjected to electrical current is covered by a network of cracks. These features are more intense from sample N2 to N3 following the severity of electrical current conditions applied. In addition, droplet-like islands in N2 show that melting occurred in certain areas, manifesting thus a local temperature increase during the treatment of the samples by electrical pulsing. For comparison, the samples conventionally annealed and etched under the same conditions are shown in Figure 5g,h. They possess a perfectly uniform microstructure revealing micron and submicron grains of the crystallization products.

An explanation can be suggested in order to rationalize the observed microstructures of the ribbons crystallized under electrical current. Crystallization in amorphous ribbons starts in certain zones (first crystallized zones), which further determine the crystallization process and evolution of the microstructure [22]. Generally, these zones could be associated either with local compositional deviations increasing the likelihood of crystallization or with thickness variations and/or surface defects of the ribbons practically unavoidable during melt-spinning. In multicomponents metallic glasses, nanoscale compositional heterogeneities were predicted by Fujita et al. [23] using molecular dynamics simulation while mesoscale (submicron) heterogeneities were experimentally observed by Caron et al. [24]. In the present work, a binary metallic glass crystallizes and shows non-uniformities in the microstructure, whose scale is tens of microns. The surface of the initial amorphous ribbon etched under the same conditions as the crystallized ribbons reveals certain features that could appear as a result of the initial surface roughness of the as-spun ribbons. The scale of these features and their random distribution coincides with the scale and distribution of non-uniformities observed in the crystallized ribbons as droplet-like featureless areas. This allows us to draw a conclusion that the thickness variations and surface defects play a significant role in determining the behavior of the ribbons during crystallization under electrical current. The fact that the same ribbons when heated conventionally show a uniform microstructure clearly indicates that the observed non-uniformities formed as a response of the alloy ribbons to the passing electrical current. The as-spun ribbons are not ideally flat and the thinner areas of the ribbons are heated up to higher temperatures relative to the average temperature of the sample such that the alloy locally melts in the corresponding regions. The areas that crystallized first reduce their resistivity and at later stages evolve lower Joule heat compared to the regions that are still amorphous. This forms a non-uniform temperature field in the sample, which also rapidly changes in time as the parameters of the electrical current change during the pulse. The non-uniform temperature field in the samples creates mechanical stresses further contributing to the microstructural development in the crystallized sample. Since the pulses used in this work are very short, the non-uniform microstructures formed in the crystallized samples and metastable phase compositions are quenched and can be later observed at room temperature.

In a practical aspect, a promising application of amorphous Ti-Cu-based alloys is brazing filler materials [25-27]. Owing to their flexibility and ductility, thin amorphous ribbons offer a convenient way of placing an alloy of a certain composition between the parts to be joined. Ti-Cu-based amorphous alloys are attractive brazing fillers with titanium playing the role of an active component capable of chemically reacting with the material of the brazed parts. When resistance brazing is conducted, the heat can be delivered locally to a well-defined region using electrical pulses. The crystallization behavior of the Ti-Cu alloys under electrical pulsing should be taken into account during selection of the brazing parameters. Joule heating of metallic glasses can be used for connecting and shaping purposes at temperatures within the supercooled liquid region of the glass [28]. If pulsed current is applied in such processes, a possibility of crystallization occurring locally needs to be considered as the presence of crystallization products can deteriorate the quality of the joint and its mechanical strength.

## Conclusions

We have studied the crystallization of Ti_33_Cu_67 _amorphous ribbons under electrical pulses of high current density. By varying the pulse parameters, different stages of crystallization were reached and quenched in the samples. Partial polymorphic crystallization resulting in the formation of 5- to 8-nm crystallites of the TiCu_2 _intermetallic in the residual amorphous matrix occurred when the current density reached 9.7 · 10^8 ^A m^-2 ^and the pulse duration was 140 μs. Samples that experienced higher current densities contained the Ti_2_Cu_3 _and TiCu_3_. The microstructures of ribbons crystallized by electrical pulsing showed evidence of local melting and solidification, and differed dramatically from those of the ribbons crystallized by conventional heating. The complete crystallization of the ribbons was accompanied by the formation of cracks due to extremely brittle behavior of the mixture of intermetallic phases while the nanocrystallized samples well retained their shape and wholeness.

## Methods

Titanium and copper (99.99%) were arc-melted in an argon atmosphere to prepare the master alloy. Ti_33_Cu_67 _ribbons were produced from the master ingots by rapid quenching of the liquid using the single roller melt-spinning technique. The thickness of the ribbons in the as-quenched sample varied from 25 to 100 μm, the width of the ribbons was 1.5 mm.

**Figure 6:**
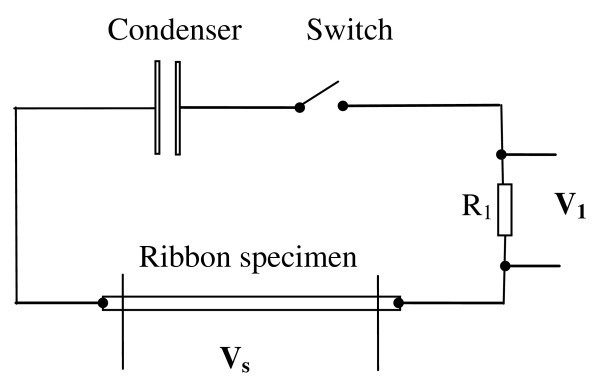
**Experimental set-up for applying electrical pulses to thin metallic glass ribbons**. R_1 _is a reference resistor.

The scheme of the experimental set-up for applying electrical pulses to thin ribbons is shown in Figure 6. No special configuration was used to provide heat sink from the ribbon pieces subjected to electrical current. The length of the ribbon samples was 20 mm.

The XRD phase analysis of the initial and crystallized ribbons was performed using a D8 ADVANCE diffractometer (Bruker) using Cu Kα radiation.

In order to prepare samples for scanning electron microscopy (SEM) observations, the initial amorphous ribbon and the crystallized samples were electrochemically etched in a HNO3-CH3OH solution containing 33 vol.% of HNO_3 _under an applied voltage of 5 V for 30 s. The SEM was performed using a Hitachi-Tabletop TM-1000 microscope (Japan).

High-resolution transmission electron microscopy (HRTEM) was performed using a JEOL-4000EX microscope operated at 400 keV. This microscope is equipped with an improved objective lens pole piece UHP-40H with Cs = 0.85 mm and characterized by 'point-to-point' resolution of 0.16 nm. Thin foils for HRTEM investigations were prepared by routine techniques including grinding and ion milling. The pieces of ribbons were cut and fixed on a standard copper holder with a slot by means of glue. Ion milling was performed using a Gatan Precision Ion Polishing System Mode 691. The total ion milling time required for the preparation of thin areas in the samples was about 2 to 2.5 h. The milling angle and the milling energy were approximately 5° and 5 keV at the first stage and 1° and 1 keV at the last stage of the ion milling process.

## References

[B1] Inoue A Stabilization of metallic supercooled liquid and bulk amorphous alloys Acta Mater 2000 48 279 306 10.1016/S1359-6454(99)00300-6

[B2] Ashby MF Greer AL Metallic glasses as structural materials Scripta Mater 2006 54 321 326 10.1016/j.scriptamat.2005.09.051

[B3] Inoue A Zhang W Tsurui T Yavari AR Greer AL Unusual room-temperature compressive plasticity in nanocrystal-toughened bulk copper-zirconium glass Phil Mag Lett 2005 85 221 229 10.1080/09500830500197724

[B4] Hajlaoui K Yavari AR Das J Vaughan G Ductilization of BMGs by optimization of nanoparticle dispersion J Alloys Comp 2007 434-435 6 9

[B5] Allia P Tiberto P Baricco M Vinai F Improved ductility of nanocrystalline Fe_73.5_Nb_3_Cu_1_Si_13.5_B_9 _obtained by direct-current joule heating Appl Phys Lett 1993 63 2759 2761 10.1063/1.110326

[B6] Gorria P Orue I Plazaola F Barandiaran JM Magnetic behavior of Fe-Nb and Fe-Zr alloys nanocrystallized by means of flash annealing J Appl Phys 1993 73 6600 6602 10.1063/1.352577

[B7] Allia P Tiberto P Baricco M Knobel M Vinai F Nanostructured materials for soft magnetic applications produced by fast dc Joule heating IEEE Trans Magnetics 1994 30 4797 4799 10.1109/20.334225

[B8] Trudeau ML Boily S Schultz R Rapid thermal annealing of Fe-based amorphous ribbons Mater Sci Forum 1996 225-227 689 694

[B9] Takemoto R Nagata M Mizubayashi H Effects of passing electric current on the elastic property of amorphous Cu_50_Zr_50 _and Cu_50_Ti_50_ Acta Mater 1996 44 2787 2795 10.1016/1359-6454(95)00378-9

[B10] Mizubayashi H Kameyama N Hao T Tanimoto H Crystallization under electropulsing suggesting a resonant collective motion of many atoms and modification of thermodynamic parameters in amophous alloys Phys Rev B 2001 64 054201-1 054201-10

[B11] Mizubayashi H Hao T Tanimoto H Low temperature crystallization of amorphous alloys under electropulsing J Non-Cryst Solids 2002 312-314 581 584

[B12] Mizubayashi H Takahashi T Nakamoto K Tanimoto H Nanocrystalline transformation and inverse transformation in metallic glasses induced by electropulsing Mater Trans 2007 48 1665 1670 10.2320/matertrans.MJ200726

[B13] LaGrange T Grummon DS Reed BW Browning ND King WE Campbell GH Strongly driven crystallization processes in a metallic glass Appl Phys Lett 2009 94 184101-1 184101-3

[B14] Girzhon VV Smolyakov AV Babich NG Semen'ko MP Effect of pulsed laser heating on the magnetic properties of the amorphous alloy Fe_76_Si_13_B_11_ The Physics of Metals and Metallography 2009 108 125 130 10.1134/S0031918X09080043

[B15] Sun H Flores KM Microstructural analysis of a laser-processed Zr-based bulk metallic glass Metall Mater Trans A 2010 41 1752 1757 10.1007/s11661-009-0151-4

[B16] Conrad H Effects of electric current on solid state phase transformations in metals Mater Sci Eng A 2000 287 227 237 10.1016/S0921-5093(00)00780-2

[B17] Knobel M Piccin R da Silva FCS Botta WJF Yavari AR Controlled crystallization of metallic glasses through Joule heating Mater Res Soc Symp Proc 2001 664 L 5.3.1 5.3.12

[B18] Holland TB Loffler JF Munir ZA Crystallization of metallic glasses under the influence of high density dc currents J Appl Phys 2004 95 2896 2899 10.1063/1.1642280

[B19] Buschow KHJ Thermal stability of amorphous Ti-Cu alloys Acta Metall 1983 31 155 160 10.1016/0001-6160(83)90075-5

[B20] Zaprianova V Raicheff R Gattef E Structure, crystallization and electrochemical corrosion behavior of amorphous Cu_66_Ti_34 _alloy Cryst Res Technol 1998 33 425 433 10.1002/(SICI)1521-4079(1998)33:3<425::AID-CRAT425>3.0.CO;2-6

[B21] Brandes EA, Brook GB Smithells Metals Reference Book 1992 7 Reed Educational and Professional Publishing Ltd

[B22] Morón C Maganto F Zato JG Garcia A Crystallization study during DC Joule heating in amorphous ribbons IEEE Trans Magnetics 2002 38 2459 2461 10.1109/TMAG.2002.803599

[B23] Fujita T Guan PF Sheng HW Inoue A Sakurai T Chen MW Coupling between chemical and dynamic heterogeneities in a multicomponent bulk metallic glass Phys Rev B 2010 81 140204 1-140204-4

[B24] Caron A Wunderlich R Gu KL Fecht HJ Structurally enhanced anelasticity in Zr-based bulk metallic glasses Scripta Mater 2011 64 946 949 10.1016/j.scriptamat.2011.01.043

[B25] Naka M Tanaka T Okamoto I Joining of silicon nitride using amorphous Cu-Ti filler metal Trans JWRI 1987 16 83 90

[B26] Nishino T Urai S Naka M Interface microstructure and strength of SiC/SiC joint brazed with Cu-Ti alloys Eng Fracture Mech 1991 40 829 836 10.1016/0013-7944(91)90240-2

[B27] Liu CF Zhang J Meng QC Zhou Y Naka M Joining of silicon nitride with a Cu_76.5_Pd_8.5_Ti_15 _filler alloy Ceramics Intl 2007 33 427 431 10.1016/j.ceramint.2005.10.016

[B28] Oliveira M Botta WJF Yavari AR Connecting, assemblage and electromechanical shaping of bulk metallic glasses Mater Trans JIM 2000 41 1501 1504

